# 

**Published:** 1884-04

**Authors:** 


					﻿Whole Number,
CCCCLXXXI.
April, 1884.
Number 4,
Vol
THE
CHICAGO
MEDICAL
Journal & Examiner.
CHICAGO:
PUBLISHED BY THE CHICAGO MEDICAL PRESS ASSOCIATION
188 Clark Street.
ISTRead the Notice of this Journal among Advertisements
				

